# Residential exposure to mold, dampness, and indoor air pollution and risk of respiratory tract infections: a study among children ages 11 and 12 in the Danish National Birth Cohort

**DOI:** 10.1007/s10654-024-01101-z

**Published:** 2024-02-23

**Authors:** Jonathan Groot, Amélie Keller, Torben Sigsgaard, Steffen Loft, Anne-Marie Nybo Andersen

**Affiliations:** 1 Section of Epidemiology, Department of Public Health, University of Copenhagen, Copenhagen, Denmark; 2 Environment, Work and Health, Department of Public Health, University of Aarhus, Aarhus, Denmark; 3 Section of Environmental Health, Department of Public Health, University of Copenhagen, Copenhagen, Denmark

**Keywords:** Indoor air pollution, Danish National Birth Cohort, Home, Built environment, Environmental epidemiology, Respiratory tract infections

## Abstract

**Background:**

The burden of respiratory tract infections (RTIs) is high in childhood. Several residential exposures may affect relative rates.

**Objectives:**

To determine risk of RTIs in children ages 11 and 12 by residential exposures.

**Methods:**

We included children in the Danish National Birth Cohort (DNBC) at ages 11 and 12. We estimated incidence risk ratios (IRR) and 95% confidence intervals (CI) for counts of RTIs within the last year by exposure to mold/dampness, gas stove usage, summer and winter candle-burning, fireplace usage, cats and dogs indoors, and farmhouse living. We also estimated IRR and 95% CI for RTIs for predicted scores of four extracted factors (‘owned house’, ‘mold and dampness’, ‘candles’, and ‘density’) from exploratory factor analyses (EFA).

**Results:**

We included 42 720 children with complete data. Mold/dampness was associated with all RTIs (common cold: IRR_adj_ 1.09[1.07, 1.12]; influenza: IRR_adj_ 1.10 [1.05, 1.15]; tonsillitis: IRR_adj_ 1.19 [1.10, 1.28]; conjunctivitis: IRR_adj_ 1.16 [1.02, 1.32]; and doctor-diagnosed pneumonia: IRR_adj_ 1.05 [0.90, 1.21]), as was the EFA factor ‘mold/dampness’ for several outcomes. Gas stove usage was associated with conjunctivitis (IRR_adj_ 1.25 [1.05, 1.49]) and with doctor-diagnosed pneumonia (IRR_adj_ 1.14 [0.93, 1.39]). Candle-burning during summer, but not winter, was associated with several RTIs, for tonsillitis in a dose-dependent fashion (increasing weekly frequencies vs. none: [IRR_adj_ 1.06 [0.98, 1.14], IRR_adj_ 1.16 [1.04, 1.30], IRR_adj_ 1.23 [1.06, 1.43], IRR_adj_ 1.29 [1.00, 1.67], and IRR_adj_ 1.41 [1.12, 1.78]).

**Conclusion:**

Residential exposures, in particular to mold and dampness and to a lesser degree to indoor combustion sources, are related to the occurrence of RTIs in children.

**Supplementary Information:**

The online version contains supplementary material available at 10.1007/s10654-024-01101-z.

## Introduction

The burden of respiratory tract infections (RTIs) is high in childhood and prevention would increase child health substantially [1]. Indoor environments are composed of numerous potentially hazardous exposures that may have an underappreciated impact on children’s health. Many of these indoor exposures have been extensively studied in relation to asthma, including but not limited to residential mold and dampness, pet allergens, secondhand smoking, carpeting and RTIs [2, 3]. The evidence on health effects related to indoor particle pollution (such as gas stove usage and incense burning) is less convincing at present [3]. Even so, a lesser focus has been on RTIs as outcomes themselves, [4] despite evidence that RTIs in infancy may increase risk of asthma [5] and evidence suggesting a similar association between mold and dampness and RTIs as to that observed for asthma [6]. 

Indoor exposures are especially relevant given that the majority of daily hours are spent indoors in Denmark and other countries in the temperate climate zone [7]. In the home, several sources of particle pollution exist, from gas stoves, to fireplaces and tobacco smoking. Additionally, there exist many microbial and allergen exposures with potential health effects, such as mold and dampness, pet allergens, livestock-related microbiota brought indoors in farmhouses, among others [8]. These are some of the dominating potentially hazardous exposures in homes in high-income countries. Mold and dampness may modestly increase risk of respiratory tract infections (RTIs) in children [9]. Use of gas stoves, especially without proper ventilation, can lead to high concentration of hazardous nitrogen dioxide and particles in the home [10, 11]. Leakage of harmful pollutants in homes outfitted with gas stoves may occur even when the appliance is not in use [10]. Findings on risk of RTIs in children living in homes with gas stoves have indicated no assocation, [12–14] but increases in chronic inflammation and non-infectious respiratory outcomes have been reported [12, 14]. Using a gas stove as a source of heat may also lead to greater risk of pneumonia, although this was not observed when using an exhaust hood while cooking [15]. An additional and underappreciated source of ultrafine and fine particles in homes, at least in Denmark, is from candle-burning [37], although studies on potential respiratory effects of candle-burning are limited to adults [16, 29]. Although adverse respiratory outcomes, including RTIs in children, have been acknowledged for exposure to combustion of solid fuels used in cooking in low- and middle-income countries [17], there has been far less focus on modern fireplaces as potential sources of indoor pollution [17]. Wood-burning can lead to large amounts of particle pollution, but both the resulting indoor pollution levels and health effects of fireplace usage in developed countries may be attenuated compared to those observed with biomass burning over open fires [17]. Although allergic sensitization and risk of asthma have been investigated to better understand risks of these in children with pet ownership, [18] the association between pets and RTIs has not been extensively investigated. Likewise, decreased rural living and increased urbanization (i.e. less ‘farming effect’) has been postulated to account for increasing incidences of allergies and asthma in high-income countries - yet the evidence for such an effect is inconclusive and the potential consequences for other immune-mediated health outcomes, such as RTIs, are complex [30].

Many of the putative hazardous indoor exposures are highly correlated, as we have previously demonstrated, [8] and potential effects of exposures to any of these may be confounded by the others. Additionally, examining mixed effects or interactions between rare and less rare exposures requires samples exceeding that of most cohorts. Although a few studies have examined certain of these exposures in terms of the risk of RTIs in children, [19] several of these exposures have not been considered as potential risk factors for RTIs and few studies have examined correlated exposures with appropriate confounder adjustment. Identifying modifiable risk factors for RTIs is important in light of the great burden of RTIs in children. The aim of this study was to examine the potential role of several distinct and related indoor home environmental exposures on symptomatic RTIs in Danish children ages 11 and 12.

## Methods

### Study population

We use data from the Danish National Birth Cohort, a birth cohort established with approximately 100 000 pregnancies in women enrolled around the year 2000 [20]. At an 11-year follow-up of the children, parents were asked to report on the indoor home environment of their children. We have previously described the many variables collected and their distributions, as well as some of their determinants [8]. In brief, built environmental factors (housing age, housing type, number of household members and square meters and ownership status), the most common determinants of gaseous and particle pollution in the home (gas stove, exhaust hood, fireplace, candles, and tobacco smoking), mold and dampness in various rooms, and pet ownership items were included. These exposures generally referenced an ‘ever’ exposure for the child (e.g. ever exposure to mold/dampness) or could reflect the current housing situation (e.g. ‘current’, same or different housing than at a given child age). The classification of ‘ever’ and ‘current’ exposures were determined by the wording of the questionnaire items, rather than analyst categorization. An example of a questionnaire item would be how often the parent burns candles during the summer-time, with the following options: never, rarely, <once a week, 1–2 times a week, 3–4 times a week, 5–6 times a week and daily (codebook for questionnaire is available online for the translated verbatim items at: https://www.dnbc.dk/data-available/11-year-follow-up). We assumed that these exposures reflect static or behavioural exposures that conditional on parental health status are likely time-invariant (e.g. farmhouse living remains the same over time and candle-burning is not changed based on other health conditions than asthma and allergies).

In the same questionnaire, the responding parent was asked to report on RTIs in the child. These were reported for the preceding year and frequencies were reported for episodes lasting three days or more. We generally assume these episodes to represent acute symptomatic illness episodes beyond the mildest infections (e.g. asymptomatic or short-duration, mildly symptomatic infections). For most outcomes, these frequencies were reported as no/never, 1–2 times, 3–4 times, 5–6 times and > 6 times. We chose to treat each of these as counts in our analyses (0, 1, 2, 3, 4). Among the diseases reported were common cold, influenza, pneumonia, tonsillitis, and conjunctivitis. In the case of common cold, ‘almost a chronic condition’ was treated as > 6 times. The number of pneumonia episodes was defined by episodes with a doctor-diagnosis and in analyses the three highest ‘counts’ were aggregated. The concurrent collection of exposures and outcomes in this study leads to a cross-sectional analysis in the cohort. Although a select number of indoor and housing environment exposures are available from the perinatal period which could be used for prospective analyses, these are limited compared to those provided at ~ 11 years.

### Ethics

The DNBC is approved by the Danish Data Protection Agency (18/04608) and the Committee on Health Research Ethics (case no. (KF) 01-471/94). Informed consent was obtained by participating mothers upon enrolment into the birth cohort.

### Variables and statistics

#### Primary analyses

In our primary analyses, we estimated incidence rate ratios (IRRs) and 95% confidence intervals (CIs) between the individual exposures of most interest from the home environment and counts of RTIs in Poisson regression models. We applied robust standard errors to account for dependent observations (i.e. multiple children from same mother (*n* = 2881 not unique observations for mother)). Exposures were parentally-reported in the 11-year follow-up questionnaire, in which all answers were framed as current or ever exposures. The exposures of interest were mold and dampness in the home ([ever exposure], yes/no), gas stove usage ([current exposure] gas stove/electric stove with or without gas oven), candle-burning during summer and winter ([current exposure] never or rarely, <1x weekly, 1–2 x/weekly, 3–4 x/weekly, 5-6x/weekly, daily), fireplace usage ([current exposure] never or rarely, <1x weekly, 1-2x/weekly, 3-4x/weekly, 5-6x/weekly, daily), dog or cat ownership ([current exposure] yes, indoors/no, outdoors), and farmhouse residency ([current exposure] other housing/farmhouse without animals/farmhouse with animals). Models were adjusted for potential covariates identified *a priori* using a directed acyclic graph (DAG) within a causal inference framework [21]. These included indoor environment variables as described above, covariates with administrative registry data (maternal education (International Standard Classification of Education (ISCED) [22] 0–2, 3–4, 5–8), equivalized household income in quartiles (1st, 2nd, 3rd, 4th) [23], maternal age at birth (< 25/25-29.9/30-34.9/≥35 years)) and a combination of registry and self-reported data ((parental asthma (yes/no), parental allergies (yes/no), parental affective disorders (yes/no), parental diabetes (yes/no) and parental cardiometabolic disorders (yes/no)) and sex of the adolescent (female/male) (see eFigures 1 and 2). The aggregate variable ‘indoor environment’ in the DAG was specified further for each model by including only those built and indoor home environment variables that were previously identified as loading on the same factors (‘owned house’, ‘mold and dampness’, ‘candles’ and ‘density’, renamed from previous publication) in exploratory factor analysis (EFA) [8]. For example, for the exposure ‘gas stove’, we adjusted for exhaust hood usage, building year, home ownership, and mold and dampness, in addition to the other covariates identified in the DAG (eFigure 1). For mold and dampness, we modelled an aggregate mold and dampness variable, since several mold and dampness variables might be inextricably linked and adjustments could lead to over-adjustments for collinear factors, when the purpose is to identify causal associations. An additional adjustment was made for the size of the dwelling for those exposures whose health effects are likely determined by the volume of indoor air (e.g. pollution sources such as candles), since the volume of indoor air may have an independent effect on risk of infections by dilution of virus particles. An explicit DAG for model assumptions of the summer candle-burning analyses is provided in supplement (eFigure 2).

#### Secondary analyses

In secondary analyses, we explored an alternative way to model these associations.

We considered the clustering of correlated variables with reduced numbers of variables, by modelling counts of RTIs by predicted scores based on previously described factors from exploratory factor analyses (EFA) [8]. This agnostic approach to identify clustered exposures was used to test whether an exploratory approach would (a) point to similar associations and (b) suggest potential relevant patterns of exposures of relevance. More details of the correlations and extraction of factors are available in our previous publication [8]. Briefly, EFA was conducted on all variables for housing and the indoor home environment, as described previously [8]. A polychoric correlation matrix was computed and factors were extracted after orthogonal rotation. We used the Kaiser criterion for factor retention, including the 4 factors with eigenvalues over 1 as shown in the screeplot (eFigure 3). Predicted scores were computed using Bartlett’s regression for the following 4 extracted factors, ‘owned house’, ‘mold and dampness’, ‘candles’, and ‘density’. Scores were subsequently divided into quartiles to account for their skewed and multimodal distributions and to make comparisons of unit-increases comparable across factor scores. Poisson models were constructed with mutual adjustments for other factor scores and the covariates identified in the DAG.

#### Sensitivity analyses

In addition to the above, we conducted a sensitivity analysis in which we restricted to children who had lived in the same home since birth, i.e. with a greater likelihood of lifelong exposure to some of the time-varying exposures we examined. We fit Poisson regression models identical to the primary analyses, but restricted to children who had lived in the same home since birth to address concerns about reverse causality and misclassification of time-varying exposures.

All statistical analyses were conducted in Stata software (StataCorp, Tx, USA).

## Results

**Fig. 1:**
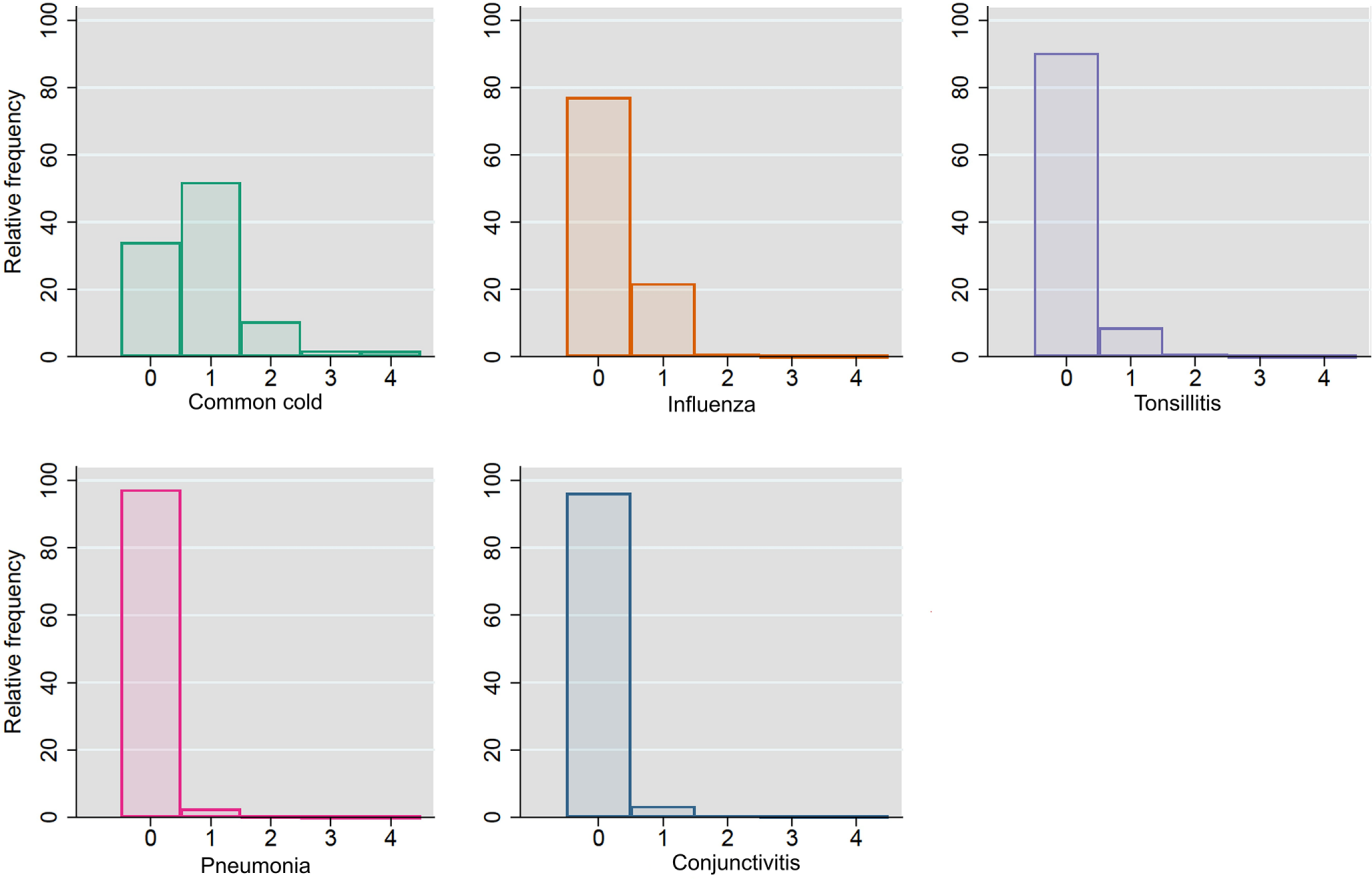
Distribution of parentally-reported respiratory tract infections in adolescent sample (*N* = 42 720) ^a^Presented and treated as counts. However, number of episodes in last year are interval if non-zero: 0 = no/never, 1 = 1–2 times, 2 = 3–4 times, 3 = 5–6 times, and 4 = > 6 times

We included 42 720 children of the approximately 96 000 children born into the DNBC, as previously described, [8] with additionally three observations excluded due to subsequent withdrawal from the cohort or missing infection data (eFigure 4). The proportions who reported having an infection lasting more than 3 days once or more within the last year or in the case of pneumonia, one or more doctor-diagnosed episodes, were: 66% for common cold, 23% for influenza, 10% for tonsillitis, 3% for pneumonia, and 4% for conjunctivitis (Fig. 1 and eTable 1). According to parental reports 20% were exposed to mold or dampness in the home, 9% to gas stove usage, 49% to some level of candle-burning during the summer, 94% to some level of candle-burning during winter, 43% to some level of fireplace usage, 51% to keeping cats and/or dogs indoors, and 12% to living in a farmhouse with or without livestock (see eTable 2).

We have extensively reported on the socioeconomic and health status determinants of the indoor home environment in the study population in a previous publication [8]. Since this sample differs only by three observations, we provide only a general summary here for simplicity and with respect to personally identifiable information. Briefly, the overwhelming majority of children in this study had mothers who had completed a medium or high education at childbirth, reflecting the generally higher socioeconomic position of participants in the DNBC. Self-reported allergies were prevalent among parents. In general, housing characteristics, such as mold and dampness, were more favorable for higher income and education groups and those without parental chronic diseases. There were a few exceptions, notably that substantive differences for candle-burning were less meaningful compared to other exposures. This sample therefore represents a higher socioeconomic position population with relatively high exposures to particle pollution from candle-burning compared to other cohorts, but lower exposures to gas stove usage, among other exposures.

### Primary analyses

**Fig. 2:**
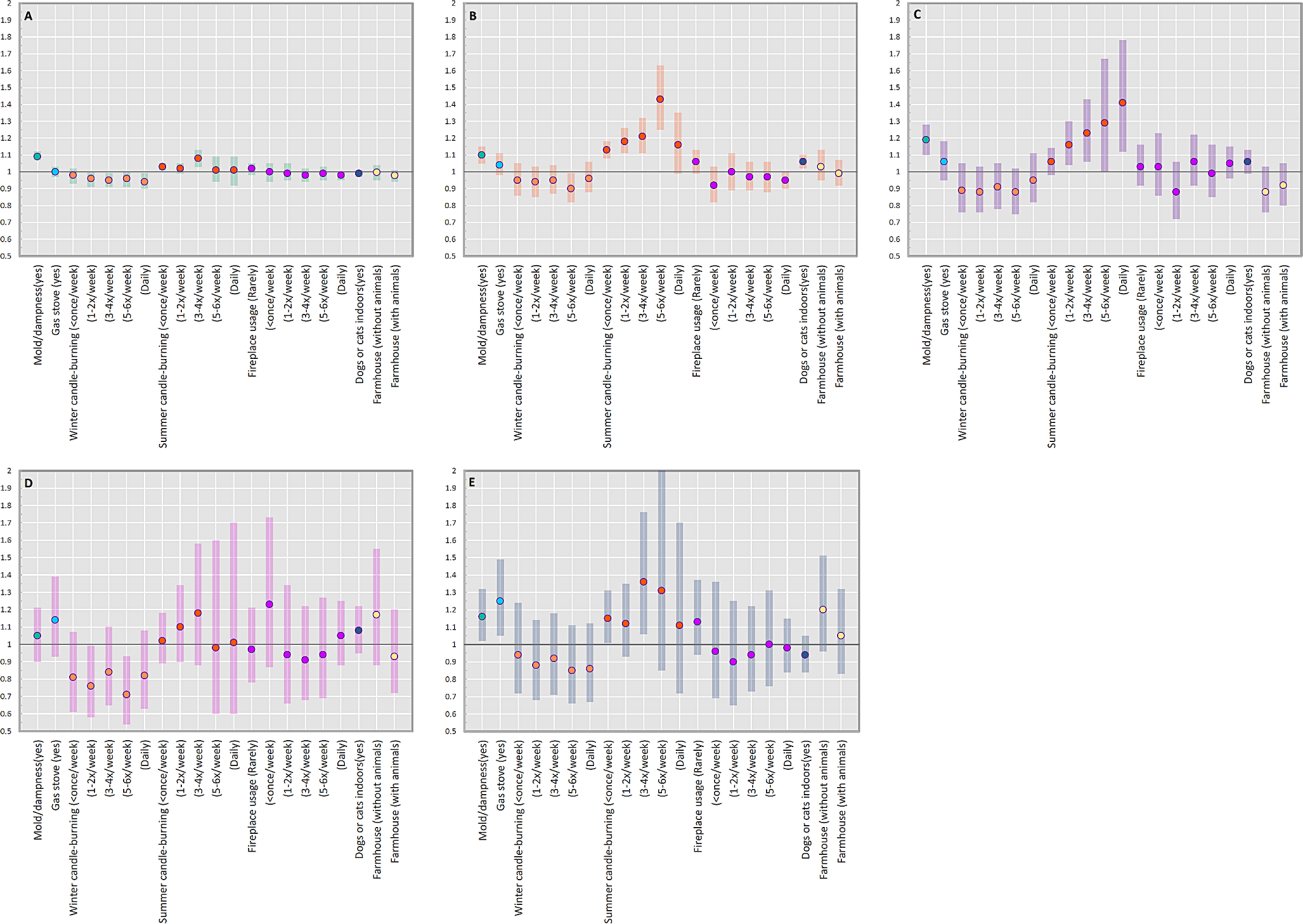
Housing and indoor home environment exposures and adjusted IRRs and 95% CIs of (**A**) common cold, (**B**) influenza, (**C**) tonsillitis, (**D**) pneumonia and (**E**) conjunctivitis in 42 720 Danish adolescents Mold/dampness (Adjusted for sex, maternal age at birth, household equivalized income at birth, maternal education at birth, parental affective disorders, parental asthma, parental allergies, parental diabetes, parental cardiometabolic disorders, year built, gas stove usage, exhaust hood usage, fireplace usage, cat and dog ownership indoors, tertiles of household density, number of rooms, number of household members, moving during study period); gas stove usage (Adjusted for sex, maternal age at birth, household equivalized income at birth, maternal education at birth, parental affective disorders, parental asthma, parental allergies, parental diabetes, parental cardiometabolic disorders, year built, mold/dampness, exhaust hood usage, fireplace usage, cat and dog ownership indoors, tertiles of household density, number of rooms, number of household members, moving during study period); winter candle-burning (Adjusted for summer candle-burning, equivalized household income at birth, maternal education at birth, parental affective disorders, parental asthma, parental allergies, parental diabetes, parental cardiometabolic disorders, maternal age at birth, sex, and dwelling size); summer candle-burning (Adjusted for winter candle-burning, equivalized household income at birth, maternal education at birth, parental affective disorders, parental asthma, parental allergies, parental diabetes, parental cardiometabolic disorders, maternal age at birth, sex, and dwelling size); fireplace usage (Adjusted for mold/dampness, gas stove usage, building year, exhaust hood usage, no. family members, household density tertiles, no. rooms, moving during childhood, equivalized household income at birth, maternal education at birth, housing type, ownership, parental affective disorders, parental asthma, parental allergies, parental diabetes, parental cardiometabolic diseases, sex, maternal age, and dwelling size); cats and dogs, indoors (Adjusted for mold/dampness, fireplace usage, exhaust hood usage, no. family member, household density tertiles, no. rooms, moving during childhood, ownership, housing type, equivalized household income at birth, maternal education at birth, parental affective disorders, parental asthma, parental allergies, parental diabetes, parental cardiometabolic disorders, sex and maternal age at birth); farmhouse (Adjusted for mold/dampness, cats/dogs, fireplace usage, exhaust hood usage, no. family members, household density tertiles, no. rooms, moving during childhood, ownership, equivalized household income, maternal education at birth, parental affective disorders, parental asthma, parental allergies, parental diabetes, parental cardiometabolic disorders, sex, maternal age at birth)

The results of our primary analyses are shown in Fig. 2. We found that mold/dampness was associated with increased rates of common colds (IRR_adj_ 1.09 [1.07, 1.12]), influenza (IRR_adj_ 1.10 [1.05, 1.15]), doctor-diagnosed pneumonia (IRR_adj_ 1.05 [0.90, 1.21]), tonsillitis (IRR_adj_ 1.19 [1.10, 1.28]), and conjunctivitis (IRR_adj_ 1.16[1.02, 1.32]) within the last year.

A small to null effect of gas stove usage was observed across most outcomes. Increased rates of 25% (IRR_adj_ 1.25 [1.05, 1.49]) with gas stove usage were observed for conjunctivitis. Associations with candle-burning were dependent on season. Candle-burning during summer was associated with increased rates of all outcomes in a nearly stepwise fashion for some (tonsillitis, increasing weekly frequencies vs. none: [IRR_adj_ 1.06 [0.98, 1.14], IRR_adj_ 1.16 [1.04, 1.30], IRR_adj_ 1.23 [1.06, 1.43], IRR_adj_ 1.29 [1.00, 1.67], and IRR_adj_ 1.41 [1.12, 1.78]). Candle-burning during the winter was associated with a minor reduction in rates across most outcomes compared to no candle-burning, but in a non-dose-dependent manner.

Keeping dogs or cats indoors was associated with either no or small increases of rates across most outcomes – no inverse associations were observed. For influenza, keeping a cat or dog indoors was associated with an increased relative rate of 6% (IRR_adj_ 1.06 [1.02, 1.10], Fig. 2 and eTable 3). Other results were not statistically significant or indicative of a strong but statistically nonsignificant effect.

**Fig. 3:**
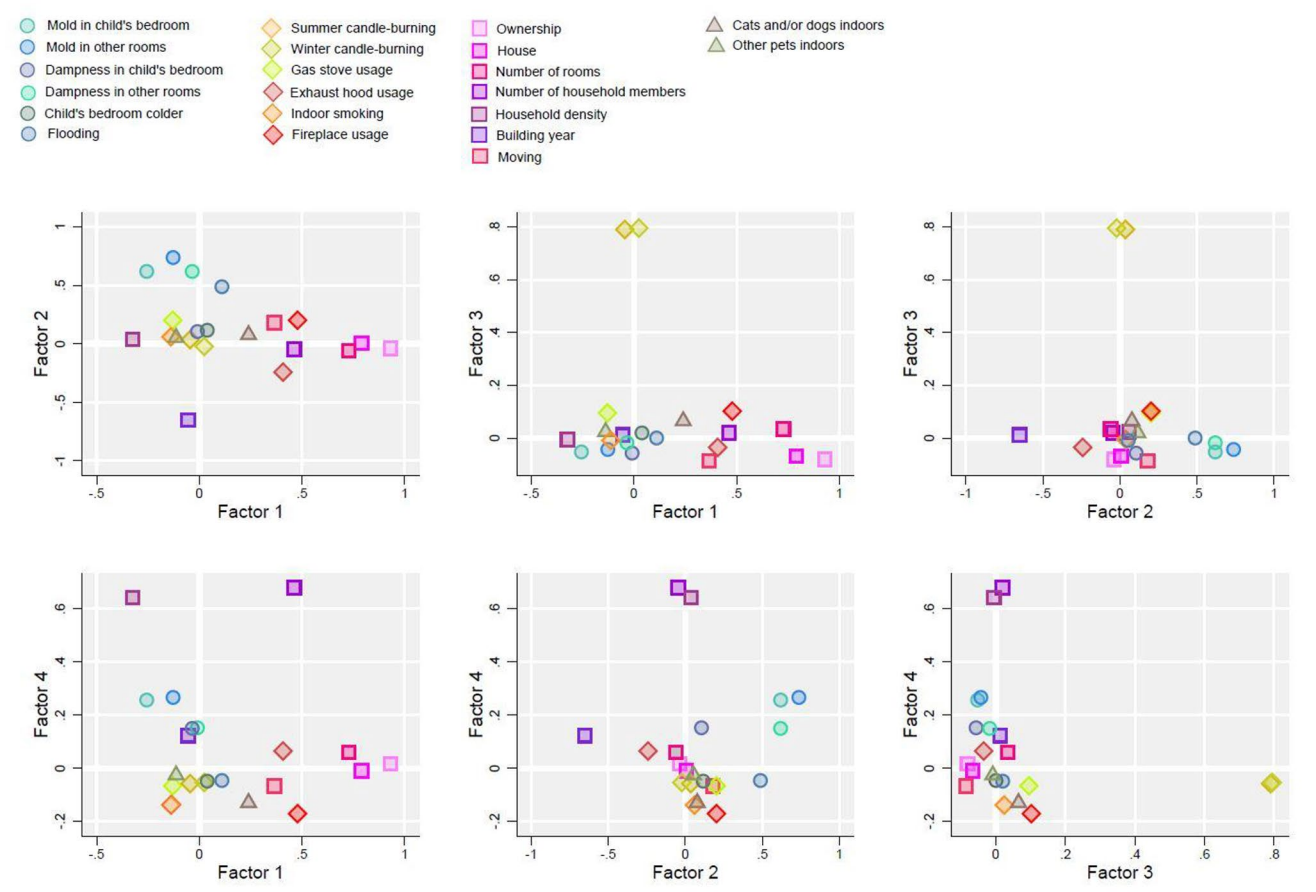
Loading plots of factor variable loadings by each factor combination *Factor loading values for each factor in the exploratory factor analysis are plotted against the factor loading values of each other factor on a two-dimensional space

Living in a farmhouse without livestock was associated with similar lower rates of common cold in the crude primary analyses as living in a farmhouse with livestock, but the associations were fully attenuated after adjustment (Fig. 2 and eTable 3).

Across all analyses, the crude and adjusted analyses did not differ substantively (see eTable 3 for comparisons between models).

### Exploratory factor analyses

**Fig. 4:**
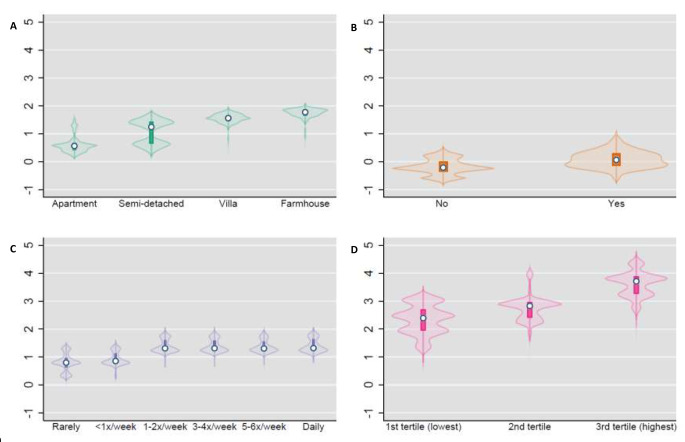
Violin plots of distribution of key indoor home environment exposures by exploratory factor analysis scores, (**A**) housing type (**B**) mold/dampness in the home (**C**) summer candle-burning (**D**) household density tertiles *The kernel density distributions of the predicted scores (y- axis) of 4 selected variables that load strongly on the four extracted factors are plotted for (**A**) Factor 1 | Owned house (**B**) Factor 2 | Mold and dampness (**C**) Factor 3 | Candles and (**D**) Factor 4 | Density

The factor loadings of each extracted factor are displayed against each other in a loading plot (Fig. 3). The first two extracted factors explaining most variance indicate that several housing factors are strongly correlated and that mold and dampness define Factor 2 (mold and dampness), but also load negatively on Factor 1 (‘owned house’). Violin plots show the kernel density distributions across categories of the predicted scores of four variables which strongly loaded on the respective extracted factors (Fig. 4). Median and interquartile ranges of EFA scores increased across categories of the selected exposure as would be expected.

**Fig. 5:**
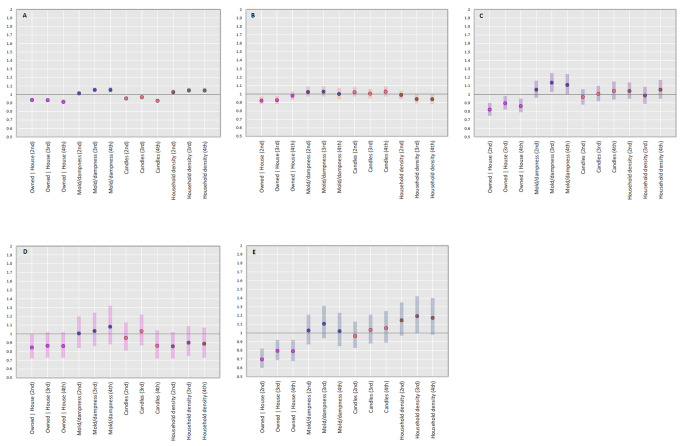
Quartiles of exploratory factor analyses predicted scores and IRRs and 95% CIs of (**A**) common cold (**B**) influenza (**C**) tonsillitis (**D**) pneumonia and (**E**) conjunctivitis in 42 720 Danish adolescents Adjusted for sex, equivalized household income at birth, maternal age at birth, maternal education at birth, parental affective disorders, parental allergies, parental asthma, parental diabetes, parental cardiometabolic disorders and mutually adjusted

Results from the Poisson regression model with quartiles of EFA predicted scores are shown in Fig. 5. Relative rates were increased for most outcomes in the highest quartile of the ‘mold/dampness’ factor: common cold (IRR_adj_ 1.05 [1.02, 1.09]), pneumonia (IRR_adj_ 1.08 [0.88, 1.32]), and tonsillitis (IRR_adj_ 1.11 [1.00, 1.24]). Likewise, relative rates were decreased for all RTIs in the ‘owned house’ factor, a factor with a strong negative factor loading for mold/dampness exposures.

Analyses for the ‘candles’ factor showed a similar but attenuated pattern as for those observed for frequencies of winter candle-burning in the primary analyses.

Analyses for the ‘mold and dampness’ factor also had high positive factor loadings for gas stove usage, so that some of the effects observed in the higher quartiles of these factor scores may be attributable to gas stove usage – conjunctivitis rates were however not substantially elevated or decreased for higher quartiles of this factor.

The EFA ‘owned house’ factor consistently indicated lower rates of most outcomes, potentially in part due to characteristics associated with or determined by farmhouse living. This factor also had negative factor loadings for mold and dampness variables and despite high factor loadings for number of household members, decreased loading on household density (Fig. 3). Except for fireplace usage, loadings for potential sources of particle pollution were also low (Fig. 3). All of these taken together suggest a more favorable exposure profile for children with higher predicted scores on the ‘owned house’ factor.

The ‘density’ factor was characterized by higher household density, dissimilar from the ‘owned house’ factor. For this factor, rates were consistently higher for common cold and conjunctivitis and lower for influenza and pneumonia. No clear dose-dependency was observed, except for common cold, for which those in the highest quartile had 5% higher rates (IRR_adj_ 1.05 [1.02, 1.08]). Most of the variance in the study sample was attributable to the first extracted factor (‘owned house’), which also had negative factor loadings for the variable household density.

### Sensitivity analysis

We repeated the primary analyses after restricting to children who grew up in the same home and had not moved. The strength and certainty of the effect estimates in these analyses were essentially the same as in the primary analyses (data not shown).

## Discussion

We found that several exposures within the housing and indoor home environment may increase the risks of RTIs in the adolescent population. In particular mold, dampness, and candle-burning, but also other housing-related indoor exposures, were associated with an increased risk of RTIs. Our results also indicate that children living in owned houses had several other favorable exposures that may decrease the risk of RTIs (i.e.increased number of rooms, lower household density and lower gas stove usage and mold and dampness). On the whole, we provide evidence for potential effects of microbial and pollutant exposures on rates of RTIs in the adolescent population.

We find associations between residential mold and dampness and RTIs, corroborating previous studies’ findings [9]. Associations between mold and dampness and most RTIs were observed regardless of model and analytic strategy. We believe this strengthens the body of evidence for the role of residential mold and dampness on respiratory infectious morbidity in children, but also suggests that at presumably lower residential exposure levels this potential effect is small. In this study we were able to address some of the research gaps in the role of mold/dampness in RTIs, by investigating RTIs specifically and adjusting for most relevant confounders [16]. Prospective data collection would increase certainty about the temporality of the observed associations. The biological mechanisms behind the findings are as of yet unclear, however, Fisk et al. suggest among several candidate mechanisms an immunomodulatory effect of mold that increases susceptibility to infection [9]. 

Our results generally indicate no increased risk of RTIs in children with fireplace usage, which may have several explanations. A recent randomized controlled trial (RCT) confirms previous reports of increased indoor pollution with wood-burning, demonstrating a dose-response effect on lower RTIs with increasing PM_2.5_ [24]. Most of the fireplaces included in the RCT were of low or medium quality. We did not have data on the quality of the fireplaces in our study, but high-grade fireplaces are expected to lead to lower indoor pollution while being a source of heating [25]. In a community intervention trial investigating changes in RTIs with substituting wood stoves for improved wood stoves or other heat sources, substantial decreases in PM_2.5_ at the neighborhood level were observed, but differences between homes with and without changed stoves were not observed [25]. This might indicate that the neighborhood ambient air pollution from wood stoves in the community is more influential than the in-home pollution. At the individual level, a similar lack of association to what we observed has been observed for pulmonary function in Danish adults, suggesting either minimal effects of particle pollution levels from fireplaces within the home on pulmonary function or potential interactions with other sources of particle pollution [26]. Most, if not all, sources of particle pollution in the home have no beneficial effect on the immune system, but fireplaces are during winter months for some a critical source of heating. The role of cold exposure on susceptibility to RTIs is potentially confounded by other factors, but if truly causal, averting cold exposure might counterbalance *some* of the negative effects of particle pollution from fireplaces [14, 27]. Additionally, high-grade fireplaces potentially enhance ventilation by mechanically extracting indoor air and replacing it with outdoor air. However, other negative health effects to residents within a residential area with particle pollution from fireplaces and the global warming effects of carbon emissions should continue to inform public health recommendations for safe and climate-friendly heating sources, even though these findings do not support a detrimental effect of the studied fireplaces on RTIs in children [28]. 

Our results suggest that candle-burning during the summer increases rates of several RTIs in a dose-response relationship, although these results must be seen in light of the inverse association and null associations for winter candle-burning. Interestingly, two studies in an adult Danish cohort also found no negative effects of candle-burning during winter on pulmonary function [26] or risk of respiratory disease events [29]. The lack of an exposure contrast, i.e. the fact that candles are used in almost all Danish households during winter time, may explain our finding [8]. 

Alternative explanations behind our findings regarding the conflicting results on candle-burning deserve elaboration. While we observed small decreased rates for winter candle-burning, the confidence intervals generally included the null and may suggest unknown confounding. At present, we suggest our findings for summer candle-burning are harder to explain in the context of unknown confounding, although this certainly cannot be excluded. The nearly linear dose-dependent relationship between summer candle-burning and RTIs may indicate a true causal effect, or a factor which closely correlates with summer candle-burning and increases rates of RTIs exists, which we have not identified in our data or literature. Although socioeconomic position is associated with candle-burning in general, in this population frequencies were substantively quite similar across strata, compared to other exposures [8]. It would be interesting to see studies replicating or refuting these associations with granular exposure data, prospective data collection, and more certain measures of infection (asymptomatic, symptomatic and severe).

While the ‘farming effect’ in the context of allergies has been proposed to be due to microbial exposures in the development of respiratory disease in children, [30] our results are also compatible with potential confounding or mediation by rurality. Children living in farmhouses are likely to live in more rural areas, go to smaller schools, and meet respiratory pathogens less frequently, compared to those in more densely populated cities. This might explain similar findings for children living in farmhouses with livestock as for those living without livestock, but does not necessarily exclude a common microbial cause that is unique to rural living. Indeed, previous studies suggest a greater microbial diversity and abundance in homes of pig and cow farmers that is not only attributable to direct microbial transfer from the farmer’s own stable [31, 32]. We would be interested to see if the common cold finding is confounded, as our primary results suggest, or points to a small, non-generalizable effect of microbial exposures unique to farm living. Interestingly, we found no inverse association with dog and cat exposure and an increased relative rate of self-reported influenza.

These results suggest that certain exposures in the home are relevant to consider in the primary prevention of RTIs in children. We report results for children at a point in life where their risks of severe RTIs are at a relatively lower point than earlier or later in life. Whether these potential environmental factors influence rates of RTIs during infancy, when the burden of RTIs is much higher, would be relevant to investigate. If these associations are causal effects, large reductions in absolute numbers of symptomatic RTIs could be achieved despite relatively small effects per exposure. We only considered the home and we could speculate that the exposome, of which we only measure a portion, could have even more meaningful impacts when addressed as a whole. The case for improved housing may include considerations of respiratory burden.

### Strengths and limitations

A major strength of our study is the large sample size with data on specific RTIs as opposed to respiratory symptoms alone – however it should be noted that these were self-reported. Many of the most relevant housing and indoor home environment exposures were also available and with sufficient granularity to investigate a dose-response relationship. We were also able to gather both self-reported and registry-based data on a large number of covariates.

Despite this, certain limitations of these data must be highlighted. First, these data are susceptible to misclassification due to misattribution of a set of symptoms to a specific illness, [33, 34] which would be less likely with a doctor-diagnosis – even then, a general practitioner (GP) who performs no diagnostic testing will not necessarily be free of misclassifying the responsible pathogen. The difficulties in distinguishing between illnesses causing influenza-like illness has recently been highlighted by Spencer et al. [34] and in a meta-analysis positivity for influenza viruses ranged only between 11 and 56% in children presenting with influenza-like illness [33]. Additionally, in our study, the one outcome which specified a doctor diagnosis (pneumonia), was also the most severe, and associations observed may not be generalizable to milder disease course. Our primary concern is misclassification with other respiratory pathogenesis unrelated to infection. This is a possibility with the self-reported nature of our outcomes, but may be less likely than misclassification between RTIs.

In a similar vein, we capture illness severity in the range most commonly experienced in this age range, but asymptomatic infections or mildly symptomatic infections lasting shorter than 3 days were not included in counts of RTIs. Asymptomatic infection with respiratory pathogens is common [35, 36]. A potential explanation for contrasting associations between self-reported common cold and influenza for the household density factor may be related to differences in effects of environment on mildly symptomatic and moderate-severe symptom RTIs. In this case, this may either illustrate true differences in determinants of disease severity or data limitations regarding our outcomes of interest.

Although most estimates are precise and with narrow CIs, consideration of several sources of bias is also warranted. We attempted to deal with confounding bias through the use of both data- and theoretically-driven selection of observable covariates. We additionally conducted exploratory analyses with an agnostic approach to exposure and covariate clustering. Several covariates of note that we did not adjust for were neighborhood characteristics like ambient air pollution and school indoor environment, since these were not available. Levels of ambient air pollution in a Danish study were inversely correlated with fireplace usage, positively correlated with gas stove usage without an exhaust hood, and not correlated with candle usage [26]. These correlations for gas stove usage and fireplace usage are likely partially related to socioeconomic position and building characteristics which we were able to adjust for. Unmeasured confounding may explain positive associations for gas stove usage and a lack of association for fireplace usage, although we expect much of this variation could be captured in the variables we adjusted for. We also did not adjust or include pollution from cooking practices themselves (e.g. interactions and independent effects of type of cooking and food type cooked). Previous research has shown that cooking itself (especially certain foods and preparation methods) is a significant source of indoor air pollution [37]. We presume that detailed data on socioeconomic position likely captures much of the neighborhood characteristics, school characteristics, and (albeit less so) food-related exposures of relevance (e.g. such as meat preparation, amount of cooking vs. frying). The role of ventilation and air exchange in RTIs is increasingly recognized, however, we did not have available data to examine natural or mechanical ventilation. There may be residual confounding due to incomplete covariate characterization, as well as unknown or unmeasured confounding variables we did not adjust for. Both negative and positive confounding might affect our results.

Since the main data in this study are of cross-sectional nature, a major concern is reverse causality. We think that the results of sensitivity analyses only including children that have lived in the same household, and therefore most likely with the same exposure pattern throughout childhood, were reassuring, as they showed a similar pattern to the main results. We believe it is rather unlikely that reverse causality would cause some of the observed associations, however – families with a high burden of RTIs in children would not be more likely to create a moist/damp home environment to compensate for this burden. We do not think that confounding by hereditary factors would explain our results, although we cannot fully exclude this possibility. It is possible that parents with respiratory diseases use an available fireplace less often, however, this mechanism would attenuate any real positive association between fireplace use and RTIs. Additionally, for parental asthma this explanation is not clearly reflected in our data. Candle-burning and gas stoves were not widely acknowledged at the time of the study as indoor pollutants, so reverse causality may be less likely for these associations. Pet ownership and farmhouse living may be related to asthmatic parents’ choices, however, this may go in both directions. To take such potential confounding into account, we adjusted for prevalent allergic and asthmatic disease in parents. Future studies with a better ascertainment of timing of exposure and changes in behavioural factors may clarify the temporal relationship between disease burden and rates of RTIs. It is an additional concern that both outcome and exposure were reported by the same respondents (parents), which could result in differential misclassification. Future studies with prospective data collection on ascertained RTIs will be important in corroborating our results.

## Conclusion

We conclude that the housing and indoor home environment may impact rates of common RTIs in 11 and 12 year olds – although better data and study designs will be needed to corroborate our findings. As found in other studies, mold and dampness are positively associated with RTIs, but this may also be affected by some of the same potential biases. Our findings suggest that candle-burning potentially increases rates of RTIs in a dose-dependent manner, although seasonal differences are in need of a good explanation. The demonstrated association between growing up on a farm and risk of immune-mediated respiratory diseases (i.e. ‘farming effect’) may be mediated or confounded by factors related to population density and in the context of RTIs may be attributable to non-livestock aspects of farming.

### Electronic supplementary material

Below is the link to the electronic supplementary material.

Supplementary Material 1
