# Supplementary material for: Residential exposure to mold, dampness, and indoor air pollution and risk of respiratory tract infections: a study among children ages 11 and 12 in the Danish National Birth Cohort
Source: Eur J Epidemiol. 2024 Feb 23;39(3):299–311. doi: 10.1007/s10654-024-01101-z (PMC10994992; doi:10.1007/s10654-024-01101-z)
Supplement: Supplementary file 1 — Supplementary Material 1 [file 10654_2024_1101_MOESM1_ESM.pdf]

**Common cold**	n	%
Never	14 542	34.0
1-2 times	22 178	51.9
3-4 times	4494	10.5
5-6 times	778	1.8
>6 times	728	1.7
**Influenza**		
Never	32 957	77.2
1-2 times	9307	21.8
3-4 times	367	0.9
5-6 times	55	0.1
>6 times	34	0.1
**Tonsillitis**		
Never	38 558	90.3
1-2 times	3702	8.7
3-4 times	351	0.8
5-6 times	70	0.2
>6 times	39	0.1
**Pneumonia**		
Never	41 585	97.3
1-2 times	1073	2.5
3 or more times	62	0.2
**Conjunctivitis**		
Never	41 139	96.3
1-2 times	1382	3.2
3-4 times	133	0.3
5-6 times	31	0.1
>6 times	35	0.1

eTable 1. Distribution of outcomes in 42 720 adolescents

**Mold/dampness**	%
No	80
Yes	20
**Gas stove**	
Electric	91
Gas stove	9
**Summer candle-burning**	
Rarely	51
<1x week	30
1-2x week	12
3-4x week	5
5-6x week	1
Daily	1
**Winter candle-burning**	
Rarely	6
<1x week	12
1-2x week	16
3-4x week	21
5-6x week	20
Daily	25
**Fireplace usage**	
No fireplace	57
Rarely	13
Weekly	14
Daily	17
**Cats and dogs indoors**	
No	49
Yes	51
**Farmhouse**	
No	88
Yes, no livestock	5
yes, with livestock	7

eTable 2. Distribution of primary exposures in 42 720 adolescents

	Common cold		Influenza		Tonsillitis		Pneumonia		Conjuctivitis	
	Crude	Adjusted	Crude	Adjusted	Crude	Adjusted	Crude	Adjusted	Crude	Adjusted
Mold/dampness										
Yes	1.11 (1.08, 1.13)	**1.09 (1.07, 1.12)**	1.12 (1.07, 1.17)	**1.10 (1.05, 1.15)**	1.22 (1.13, 1.32)	**1.19 (1.10, 1.28)**	1.07 (0.92, 1.23)	**1.05 (0.90, 1.21)**	1.22 (1.07, 1.38)	**1.16 (1.02, 1.32)**
Gas stove										
Yes	1.00 (0.97, 1.03)	**1.00 (0.97, 1.03)**	1.03 (0.97, 1.10)	**1.04 (0.98, 1.11)**	1.07 (0.96, 1.19)	**1.06 (0.95, 1.18)**	1.14 (0.93, 1.38)	**1.14 (0.93, 1.39)**	1.31 (1.11, 1.56)	**1.25 (1.05, 1.49)**
Summer candle-burning										
<1x week	1.01 (0.99, 1.03)	**1.03 (1.01, 1.05)**	1.11 (1.07, 1.16)	**1.13 (1.08, 1.18)**	1.04 (0.97, 1.12)	**1.06 (0.98, 1.14)**	0.98 (0.85, 1.12)	**1.02 (0.89, 1.18)**	1.11 (0.98, 1.26)	**1.15 (1.01, 1.31)**
1-2x week	1.01 (0.98, 1.04)	**1.02 (0.99, 1.05)**	1.17 (1.11, 1.24)	**1.18 (1.11, 1.26)**	1.18 (1.07, 1.30)	**1.16 (1.04, 1.30)**	1.06 (0.88, 1.27)	**1.10 (0.90, 1.34)**	1.08 (0.91, 1.28)	**1.12 (0.93, 1.35)**
3-4x week	1.07 (1.02, 1.11)	**1.08 (1.03, 1.13)**	1.22 (1.13, 1.33)	**1.21 (1.11, 1.32)**	1.28 (1.11, 1.46)	**1.23 (1.06, 1.43)**	1.17 (0.89, 1.53)	**1.18 (0.88, 1.58)**	1.32 (1.04, 1.67)	**1.36 (1.06, 1.76)**
5-6x week	1.02 (0.94, 1.10)	**1.01 (0.94, 1.09)**	1.48 (1.30, 1.68)	**1.43 (1.25, 1.63)**	1.40 (1.09, 1.80)	**1.29 (1.00, 1.67)**	1.00 (0.62, 1.61)	**0.98 (0.60, 1.60)**	1.31 (0.85, 1.99)	**1.31 (0.85, 2.02)**
Daily	0.99 (0.91, 1.07)	**1.01 (0.92, 1.09)**	1.19 (1.02, 1.38)	**1.16 (0.99, 1.35)**	1.51 (1.21, 1.88)	**1.41 (1.12, 1.78)**	1.06 (0.64, 1.75)	**1.01 (0.60, 1.70)**	1.08 (0.72, 1.64)	**1.11 (0.72, 1.70)**
Winter candle-burning										
<1x week	0.97 (0.92, 1.02)	**0.98 (0.93, 1.02)**	0.96 (0.87, 1.06)	**0.95 (0.86, 1.05)**	0.88 (0.75, 1.03)	**0.89 (0.76, 1.05)**	0.78 (0.58, 1.03)	**0.81 (0.61, 1.07)**	0.94 (0.72, 1.23)	**0.94 (0.72, 1.24)**
1-2x week	0.96 (0.91, 1.00)	**0.96 (0.91, 1.00)**	0.97 (0.88, 1.06)	**0.94 (0.85, 1.03)**	0.88 (0.76, 1.02)	**0.88 (0.76, 1.03)**	0.73 (0.56, 0.95)	**0.76 (0.58, 0.99)**	0.91 (0.70, 1.17)	**0.88 (0.68, 1.14)**
3-4x week	0.95 (0.91, 1.00)	**0.95 (0.91, 0.99)**	1.00 (0.91, 1.09)	**0.95 (0.87, 1.04)**	0.92 (0.80, 1.07)	**0.91 (0.78, 1.05)**	0.82 (0.64, 1.06)	**0.84 (0.65, 1.10)**	0.97 (0.75, 1.23)	**0.92 (0.71, 1.18)**
5-6x week	0.97 (0.92, 1.01)	**0.96 (0.91, 1.00)**	0.97 (0.89, 1.06)	**0.90 (0.82, 0.99)**	0.92 (0.79, 1.06)	**0.88 (0.75, 1.02)**	0.71 (0.54, 0.92)	**0.71 (0.54, 0.93)**	0.92 (0.72, 1.18)	**0.85 (0.66, 1.11)**
Daily	0.96 (0.92, 1.00)	**0.94 (0.90, 0.99)**	1.08 (0.99, 1.18)	**0.96 (0.88, 1.06)**	1.05 (0.91, 1.20)	**0.95 (0.82, 1.11)**	0.83 (0.65, 1.07)	**0.82 (0.63, 1.08)**	0.98 (0.77, 1.24)	**0.86 (0.67, 1.12)**
Fireplace usage										
Rarely	0.99 (0.95, 1.02)	**1.02 (0.98, 1.05)**	1.03 (0.97, 1.09)	**1.06 (0.99, 1.13)**	0.97 (0.86, 1.08)	**1.03 (0.92, 1.16)**	0.87 (0.70, 1.07)	**0.97 (0.78, 1.21)**	1.06 (0.88, 1.27)	**1.13 (0.94, 1.37)**
<1x week	0.95 (0.90, 1.00)	**1.00 (0.94, 1.05)**	0.86 (0.77, 0.96)	**0.92 (0.82, 1.03)**	0.91 (0.76, 1.09)	**1.03 (0.86, 1.23)**	1.04 (0.75, 1.45)	**1.23 (0.87, 1.73)**	0.85 (0.61, 1.19)	**0.96 (0.69, 1.36)**
1-2x week	0.95 (0.91, 1.00)	**0.99 (0.95, 1.05)**	0.94 (0.84, 1.05)	**1.00 (0.89, 1.11)**	0.78 (0.64, 0.94)	**0.88 (0.72, 1.06)**	0.80 (0.56, 1.13)	**0.94 (0.66, 1.34)**	0.80 (0.58, 1.10)	**0.90 (0.65, 1.25)**
3-4x week	0.94 (0.90, 0.97)	**0.98 (0.94, 1.02)**	0.92 (0.84, 1.00)	**0.97 (0.89, 1.06)**	0.96 (0.83, 1.10)	**1.06 (0.92, 1.22)**	0.77 (0.58, 1.03)	**0.91 (0.68, 1.22)**	0.84 (0.65, 1.08)	**0.94 (0.73, 1.22)**
5-6x week	0.95 (0.91, 0.99)	**0.99 (0.95, 1.03)**	0.92 (0.84, 1.01)	**0.97 (0.88, 1.06)**	0.90 (0.77, 1.06)	**0.99 (0.85, 1.16)**	0.80 (0.59, 1.09)	**0.94 (0.69, 1.27)**	0.89 (0.68, 1.16)	**1.00 (0.76, 1.31)**
Daily	0.95 (0.92, 0.97)	**0.98 (0.95, 1.00)**	0.92 (0.87, 0.97)	**0.95 (0.90, 1.00)**	0.98 (0.90, 1.07)	**1.05 (0.96, 1.15)**	0.94 (0.80, 1.11)	**1.05 (0.88, 1.25)**	0.90 (0.77, 1.05)	**0.98 (0.84, 1.15)**
Cats or dogs										
Yes	0.98 (0.96, 1.00)	**0.99 (0.97, 1.01)**	1.07 (1.03, 1.11)	**1.06 (1.02, 1.10)**	1.05 (0.98, 1.12)	**1.06 (0.99, 1.13)**	1.05 (0.93, 1.18)	**1.08 (0.95, 1.22)**	0.95 (0.86, 1.06)	**0.94 (0.84, 1.05)**
Farmhouse										
Yes, without livestock	0.96 (0.91, 1.00)	**1.00 (0.95, 1.04)**	1.00 (0.92, 1.09)	**1.03 (0.95, 1.13)**	0.85 (0.73, 0.99)	**0.88 (0.76, 1.03)**	1.11 (0.84, 1.46)	**1.17 (0.88, 1.55)**	1.16 (0.92, 1.45)	**1.20 (0.96, 1.51)**
Yes, with livestock	0.94 (0.90, 0.97)	**0.98 (0.94, 1.01)**	0.97 (0.90, 1.04)	**0.99 (0.92, 1.07)**	0.90 (0.78, 1.02)	**0.92 (0.80, 1.05)**	0.88 (0.69, 1.13)	**0.93 (0.72, 1.20)**	1.02 (0.82, 1.27)	**1.05 (0.83, 1.32)**

eTable 3. Housing and indoor home environment exposures and crude and adjusted IRRs and 95 % CIs of A) common cold, B) influenza, C) tonsillitis, D) pneumonia and E) conjunctivitis in 42 720 adolescents

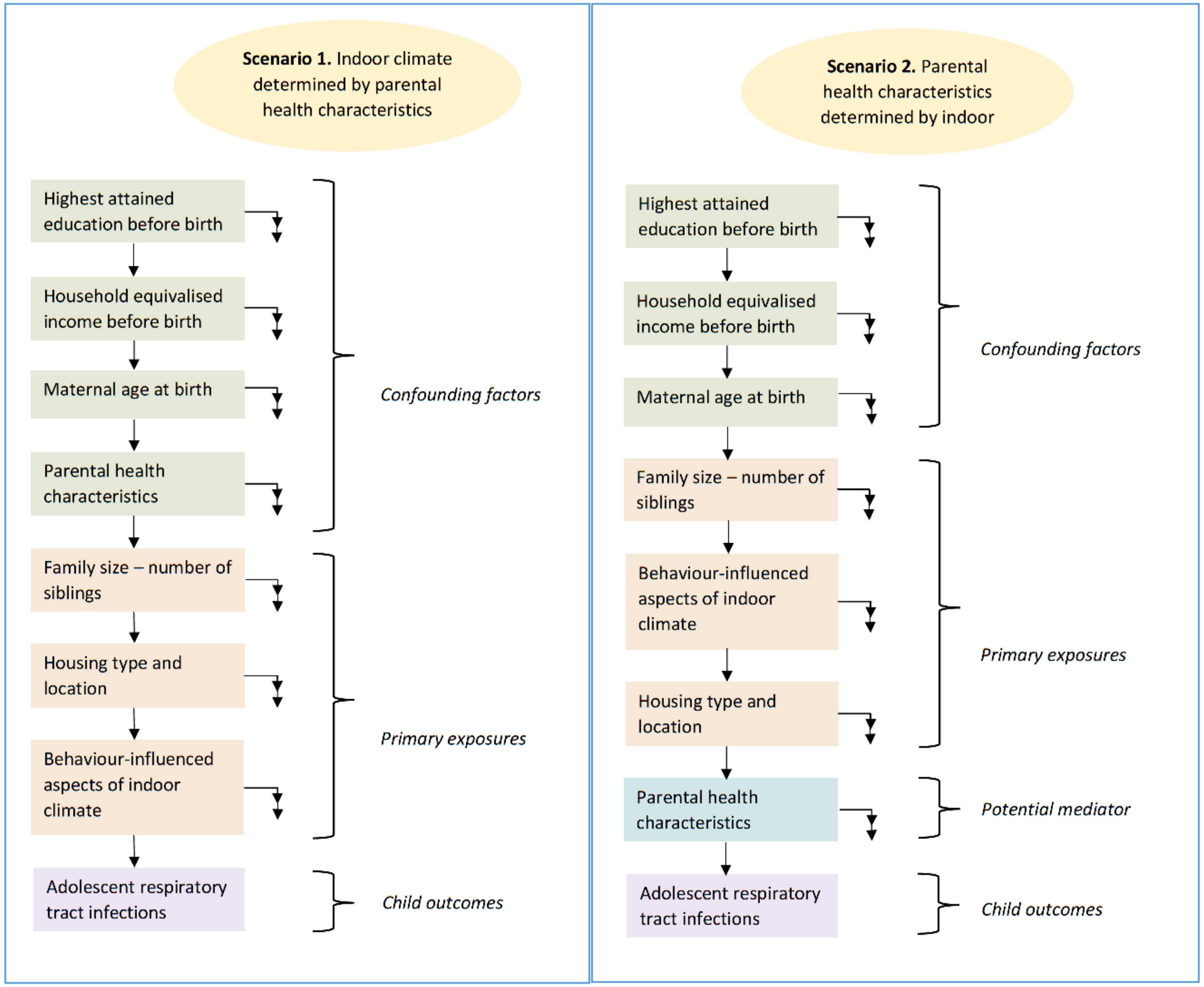

eFigure 1. Saturated directed acyclic graph

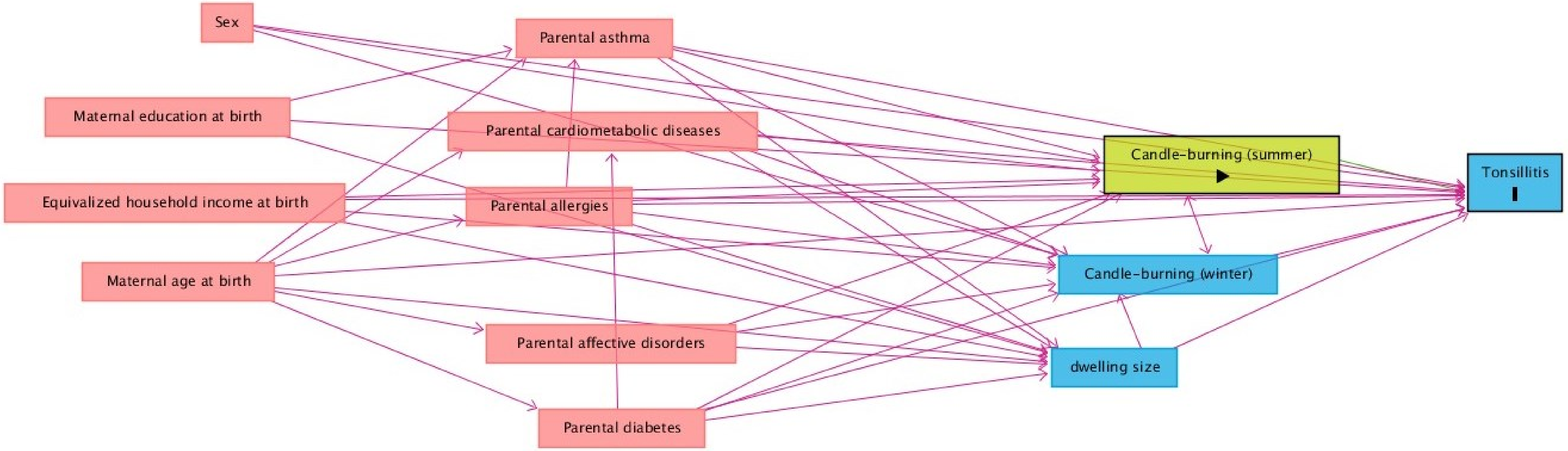

eFigure 2. Directed acyclic graph demonstrating model assumptions for analyses of summer candle-burning

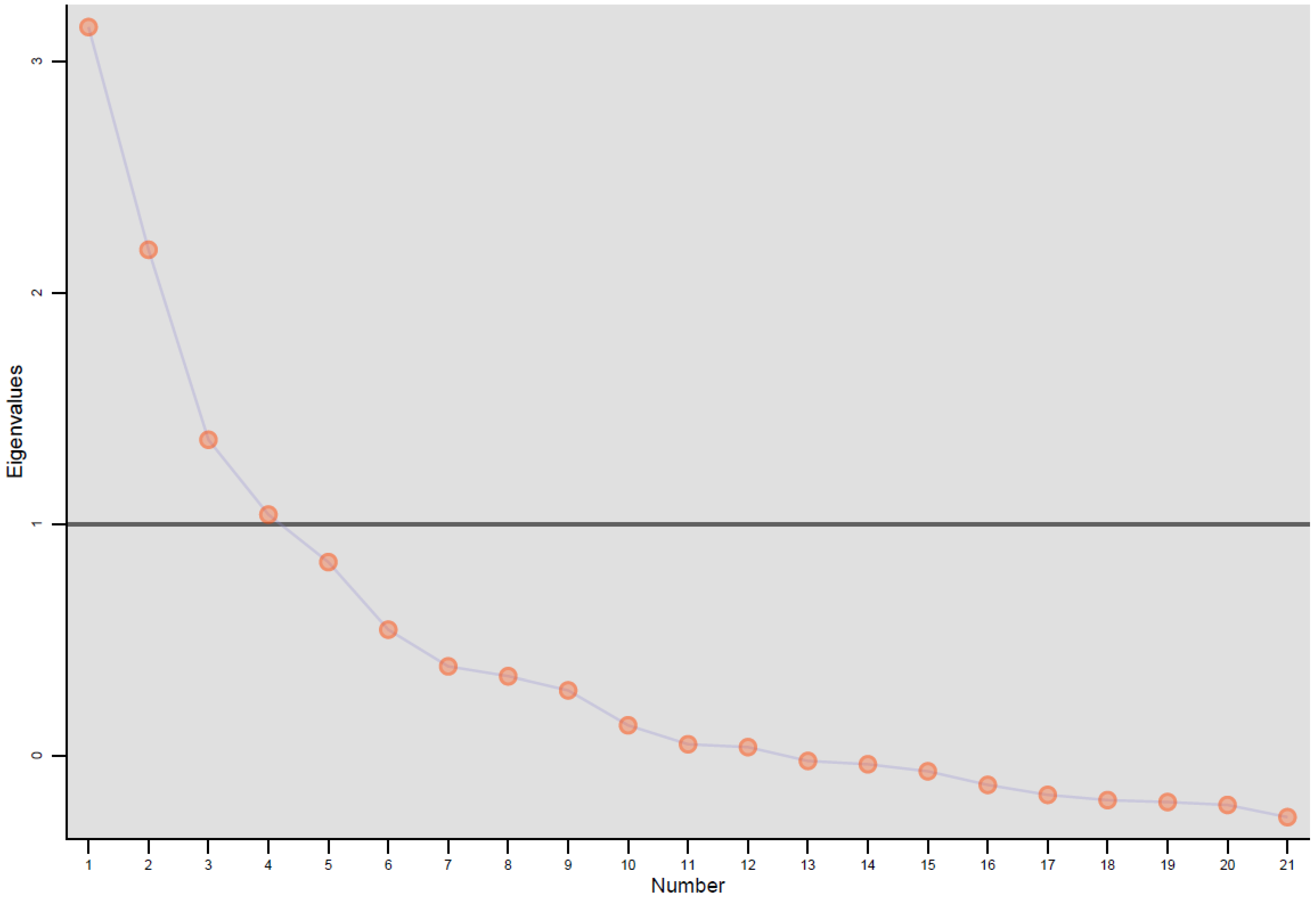

eFigure 3. Scree plot of factor retention

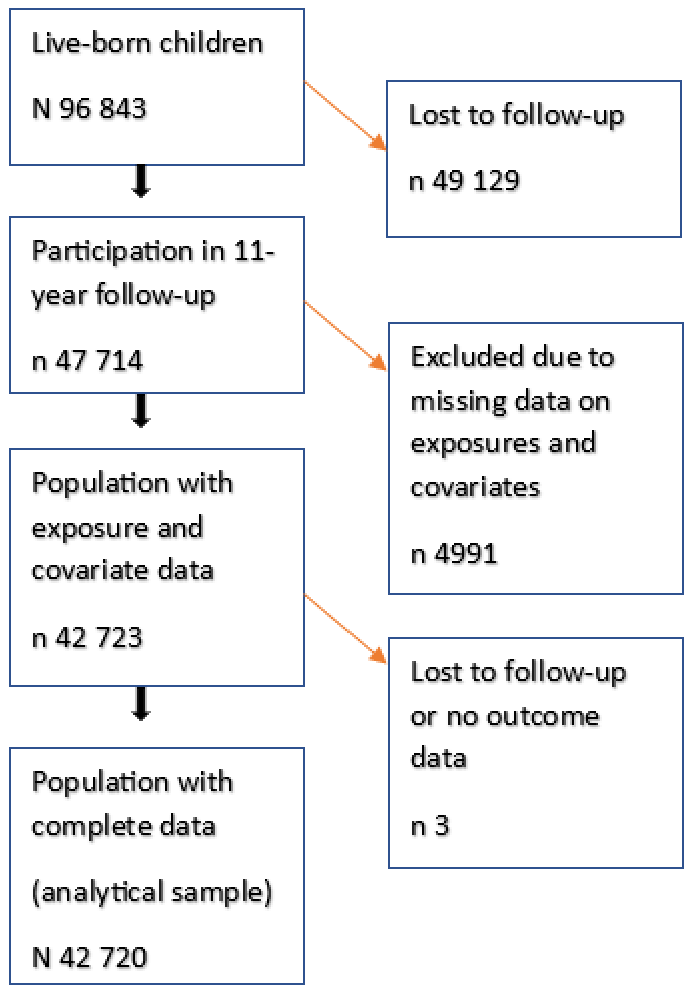

eFigure 4. Flow chart of participation
